# Comparison of Epstein-Barr virus and Kaposi’s sarcoma-associated herpesvirus viral load in peripheral blood mononuclear cells and oral fluids of HIV-negative individuals aged 3 to 89 years from Uganda

**DOI:** 10.21203/rs.3.rs-2613771/v1

**Published:** 2023-03-02

**Authors:** Angela Nalwoga, Vickie Marshall, Wendell Miley, Nazzarena Labo, Denise Whitby, Robert Newton, Rosemary Rochford

**Affiliations:** University of Colorado, Anschutz Medical Campus; Leidos Biomedical Research, Inc, Frederick National Laboratory for Cancer Research; Leidos Biomedical Research, Inc, Frederick National Laboratory for Cancer Research; Leidos Biomedical Research, Inc, Frederick National Laboratory for Cancer Research; Leidos Biomedical Research, Inc, Frederick National Laboratory for Cancer Research; University of York; University of Colorado, Anschutz Medical Campus

**Keywords:** Epstein-Barr virus, Kaposi’s sarcoma-associated herpesvirus, viral load, HIV uninfected individuals, across the age span, Uganda

## Abstract

We previously found that age, sex, and malaria were associated with KSHV viral load in individuals from Uganda. In this study, we have evaluated factors associated with presence of EBV DNA in blood and oral fluids among the same individuals, using the same biological samples. Overall, 74% of oral fluids samples and 46% of PBMCs had detectable EBV, compared to 24% and 11% for KSHV respectively Individuals with EBV in PBMCs were more likely to have KSHV in PBMCs (P=0.016). The peak age for detection of EBV in oral fluids was 3–5 years while that of KSHV was 6–12 years. In PBMCs, the peak age for detection of EBV was 66+ years and KSHV was 3–5 years. Individuals with malaria had higher levels of EBV in PBMCs compared to malaria-negative individuals (P=0.002). In summary, our results show that younger age and malaria are associated with higher levels of EBV and KSHV in PBMCs suggesting malaria impacts immunity to EBV and KSHV.

## Introduction

Among the known human herpesviruses, the gamma-herpesviruses Epstein-Barr virus (EBV) and Kaposi’s sarcoma-associated herpesvirus (KSHV) are carcinogenic [1]. EBV is causally associated with Burkitt’s lymphoma, certain Hodgkin’s and non-Hodgkin’s lymphomas, nasopharyngeal and gastric carcinomas, while KSHV is the cause of Kaposi’s sarcoma, multicentric Castleman’s disease (MCD) and primary effusion lymphoma (PEL) [2, 3]. Of these malignancies, PEL cells are infected with both EBV (~80%) and KSHV (100%) [4]. In sub-Saharan Africa, both EBV and KSHV infections are very common [5]. Infection with both viruses occurs in childhood, with EBV infection occurring earlier than KSHV [6, 7]. Both EBV and KSHV have been shown to infect and establish latency in several B cell subsets [8, 9]. However, the interaction between the two viruses has not been extensively studied ex vivo.

Lytic replication allows the dissemination of both gamma-herpesviruses to uninfected cells and is involved in tumorigenesis [10, 11]. Viral reactivation leads to the expression of lytic genes resulting in viral progeny assembly and egress [12–14]. Higher EBV or KSHV viral load in PBMCs is associated with disease [15–18]. Viral reactivation occur in associated diseases as well. Viral reactivation can occur in the context of immune suppression either caused by HIV infection or immunosuppressive drugs following organ transplant [19]. However, in HIV-uninfected individuals and in individuals not on immunosuppressive drugs, the causes of viral reactivation are not well studied. Infection with *P. falciparum* is one of the factors we and others have shown to be associated with viral reactivation of both EBV [20] and KSHV [21, 22]. The relationship between *P. falciparum* and EBV leading to Burkitt’s lymphoma has been documented [23].

We have previously shown the pattern of KSHV viral load in oral fluids and PBMCs and associated risk factors across a wide age range in HIV-negative individuals from a KSHV endemic area [24]. Furthermore, in a Cameroon KS case-control study, we have shown EBV and KSHV viral load interactions in PBMCs and oral fluids [25]. However, the Cameroon KS study included only adults some of whom were HIV infected and/or had Kaposi’s sarcoma. Here we are showing the pattern of EBV and KSHV viral load in PBMCs and oral fluids and associated risk factors across a wide (3 – 89 years old) age range in HIV-negative individuals from a KSHV/EBV endemic area.

## Methods

### Study design and population

As reported previously [24], this work was carried out within a rural African cohort, the General Population Cohort (GPC). The GPC is a community-based cohort of about 22,000 people in 25 adjacent villages in southwestern Uganda. It was established in 1989 to investigate the epidemiology of HIV; participants from the GPC have been followed ever since. The seroprevalence of KSHV is >90% in adults [26]. Between July 2017 and November 2017, we nested a cross-sectional study within the GPC enrolling 975 KSHV seropositive (tested previously [27]), HIV-negative individuals aged three to eighty-nine years. Participants were selected randomly after stratification for age and sex. Blood, stool and oral fluids samples were collected from these individuals. Socio-demographic data were collected using standard questionnaires. DNA was extracted from 2 million PBMCs collected and oral fluids pellets. This DNA was used to quantify both KSHV and EBV.

Peripheral blood mononuclear cells (PBMC) and plasma were obtained from the blood for immunological and virological analyses. Study participants were instructed to rinse with 5mL of Listerine mouthwash, and collect the resulting fluid in a polypropylene tube. Aliquots (of 1mL each) of oral fluids were spun at 13,000rcf for 10 minutes to form oral fluids pellets. Thereafter the supernatant was removed and the oral fluids pellet was stored at −80°C. A pellet of two million PBMCs and oral fluids pellets were processed for DNA extraction using a QIAamp blood kit (Qiagen, Valencia, CA), following the manufacturer’s instructions.

### EBV real-time PCR

Using DNA extracted previously [27], EBV DNA was quantified in PBMCs and oral fluids from 833 individuals with KSHV viral load data [24]. EBV viral load was quantified using real-time PCR. EBV DNA was amplified using primers (Balf5 EBV forward: 5’ – CGG AAG CCC TCT GGA CTT C – 3’, - Balf5 EBV reverse: 5’ – CCC TGT TTA TCC GAT GGA ATG – 3’) and probe (Balf5 EBV Probe: 5’ - /56-FAM/TGT ACA CGC ACG AGA AAT GCG CCT/3BHQ_1/ - 3’) previously reported to be specific to the Balf5 gene [6, 28]. Additionally, B-Actin was amplified in the same sample as an internal positive control using primers (B-Actin forward: 5’ – TCA CCC ACA CTG TGC CCA TCT ACG A – 3’, B-Actin reverse: 5’ – CAG CGG AAC CGC TCA TTG CCA ATG G – 3’) and probe (B-Actin Probe: 5’ - /5HEX/ATG CCC TCC CCC ATG CCA TCC TGC GT/3BHQ_1/ - 3’) as previously reported [29].

### KSHV and EBV serology

IgG antibody levels were quantified in plasma using ELISA and a multiplex bead-based assay as previously described [27, 30]. K8.1 and LANA/ORF73 recombinant proteins were used to quantify IgG by ELISA. Seropositivity was defined as reactivity to either K8.1 or ORF73 proteins. Each ELISA plate contained three positive and three negative control sera. The negative control sera were used to set a cut-off value on each plate as previously reported [31]. DNA was extracted from oral fluids and PBMCs of seropositive individuals. Twenty-five KSHV recombinant proteins including ORF73, K10.5, K5, K14, ORF6, ORF11, ORF55, ORF50, ORF60, K3, ORF38, ORF52, ORF59, ORF65, ORF61, ORF18, K11, K8.1, ORF19, ORF25, ORF26, ORF33, ORF37, ORF44 and ORF63 were included in the multiplex bead assay panel with three EBV proteins (EBNA-1, VCA and EA).

### Statistical analysis

Statistical analysis was carried out using STATA version 13 (StataCorp, College Station, Texas USA). Graphs were drawn using STATA and GraphPad Prism version 8. Viral load levels were log_10_ transformed. First, risk factors associated with viral DNA detection (as a categorical outcome variable) in oral fluids and PBMCs, separately, were obtained using logistic regression modelling. Thereafter, risk factors associated with increasing levels of viral DNA (as a continuous outcome variable) in oral fluids and PBMCs, separately, were determined using linear regression modelling. Chi^2^ test, student T-test, Kruskal Wallis test and one-way ANOVA were used for crude analysis. The false discovery rate (FDR) was used to correct multiple comparisons of antibody data.

## Results

**Figure 1:**
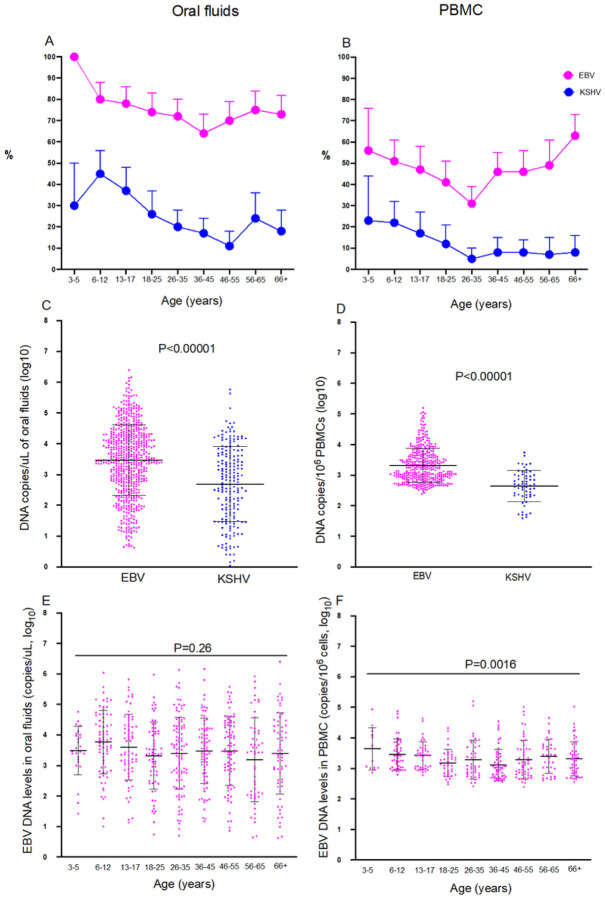
EBV and KSHV in oral fluids and PBMCs. A: proportion of individuals with EBV or KSHV in oral fluids; B proportion of those with EBV or KSHV in PBMCs; C: EBV and KSHV DNA levels in oral fluids; D: EBV and KSHV DNA levels in PBMCs. EBV viral load in oral fluids (E) and PBMCs (F) in each age group. EBV and KSHV Viral load quantified using qPCR. Graphs were drawn in GraphPad Prism version 8. Mean and standard deviation are shown in C and D. error bars represent 95% Confidence intervals in A and B. Mean and SD are shown in C, D, E and F. The student T-test (C, D) and Kruskal Wallis test (E, F) were used to obtaining P values

Characteristics of the participants included are shown in Table 1. The proportion of individuals with detectable EBV DNA in oral fluids was 74% compared to 24% for KSHV. The median EBV viral load (VL) in oral fluids were 3,364 copies/uL while KSHV VL was 401 copies/uL (Table 1). Prevalence of shedding in oral fluids varied with age: all children aged 3–5 years had EBV in oral fluids whereas adults aged 36–45 years had the lowest proportion (72%). For KSHV, the highest proportion with KSHV DNA was among 6–12-year-olds (30%) whereas adults aged 46–55 years old had the lowest (11%) The patterns of KSHV and EBV shedding with age were similar (Figure 1A).

As found in a study from Cameroon [25], the proportion of individuals with either EBV or KSHV DNA in PBMCs was much lower than the proportion of individuals with either virus in oral fluids (Figure 1B). Similarly, levels of EBV (median 1566 copies/10^6^ cells) were much higher than levels of KSHV (median 203 copies/10^6^ cells) DNA in PBMCs P<0.00001 (Figure 1D & Table 1). Overall, 46% of individuals tested had EBV DNA in PBMCs while 11% had KSHV. For both EBV (56%) and KSHV (23%), children aged 3–5 years had the highest (KSHV) and second highest (EBV) proportions of the virus in PBMCs, while adults aged 26–35 years old had the lowest proportions (EBV: 31%; KSHV: 5% (Figure 1B). Adults over 66 had the highest proportions of EBV in PBMCs (63% Figure 1B). levels of EBV DNA in oral fluids were higher than levels of KSHV DNA in oral fluids (Figure 1C). Children aged 6–12 years had the highest EBV viral load levels in oral fluids, but otherwise, these did not change much across age (Figure 1E & F).

**Figure 2:**
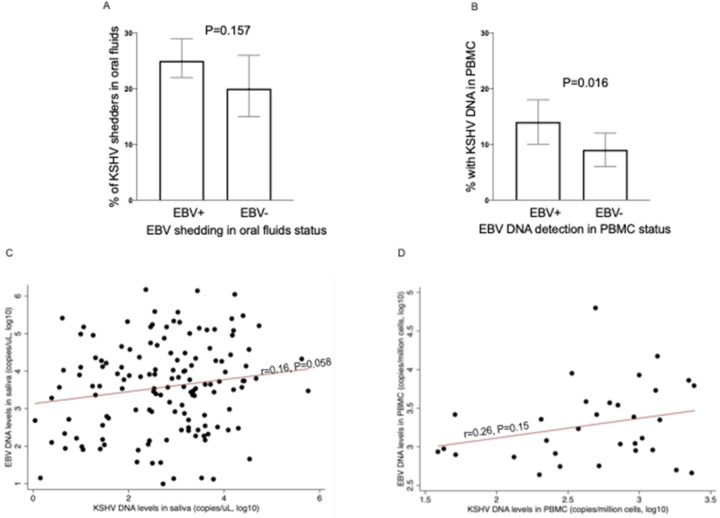
Relationship between KSHV and EBV viral load in oral fluids or PBMCs A: proportion of individuals with KSHV in oral fluids among those with or without EBV in oral fluids; B proportion of those with KSHV in PBMCs among those with or without EBV in PBMCs; C: correlation between EBV and KSHV DNA levels in oral fluids; D: correlation between EBV and KSHV DNA levels in PBMCs; D: EBV DNA levels in EBV and KSHV Viral load quantified using qPCR. Graphs were drawn in GraphPad Prism version 8 (A & B) and STATA version 13 (C & D). P values and correlation coefficient obtained in STATA version 13. The chi^2^ test is used in A and B.

The proportion of individuals with KSHV DNA in oral fluids did not differ between those with and without EBV DNA in oral fluids (Figure 2A). However, despite the lower prevalence of either virus in PBMCs compared to oral fluids, the proportion of individuals with KSHV DNA in PBMCS was higher among individuals with EBV DNA in PBMCs (14% Vs 9%, P=0.016) Figure 2B. Both in oral fluids (Figure 2C) and PBMCs (Figure 2D), EBV and KSHV DNA levels were positively correlated, although this didn’t reach statistical significance.

**Figure 3:**
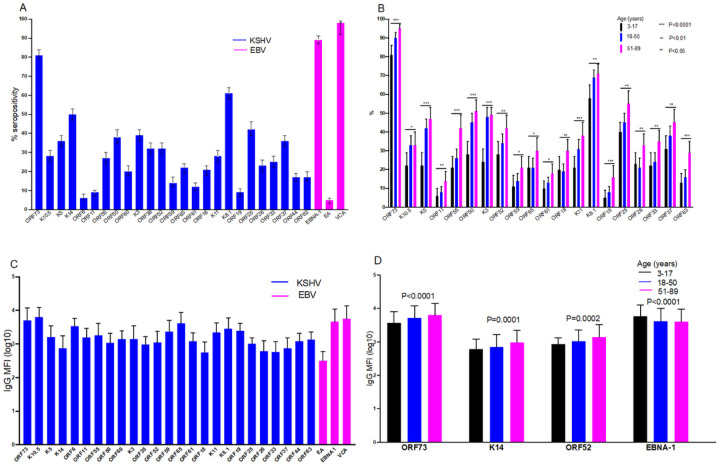
IgG responses to KSHV and EBV proteins. A: proportion of individuals with a seropositive response to KSHV/EBV proteins. B: proportion of individuals with a seropositive response to KSHV proteins by age group. C: mean IgG median fluorescent intensities-MFI to KSHV/EBV proteins. D: mean IgG MFI to KSHV proteins by age group. Error bars represent standard deviations (C & D) or 95% confidence intervals (A & B). P-value obtained using chi^2^ test (B) or one-way ANOVA (D) in STATA version 13. Graphs were drawn using GraphPad Prism version 8.

**Figure 4:**
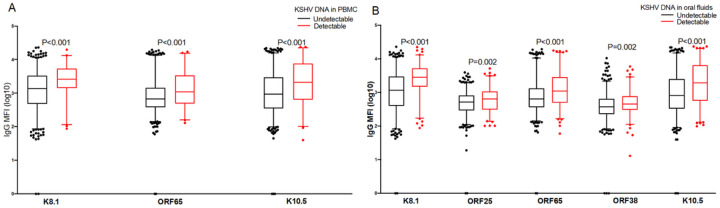
Relationship between viral load and antibody responses to KSHV. P-value obtained using a student T-test in STATA version13. Graphs were drawn in GraphPad Prism version 8.

Among the 25 KSHV and three EBV proteins used to detect IgG antibody levels in plasma, the majority of the individuals tested responded to ORF73 for KSHV and VCA for EBV (Figure 3A). The proportion of individuals responding to KSHV ORF73, K10.5, K5, ORF11, ORF55, ORF50, K3, ORF52, ORF59, ORF65, ORF61, ORF18, K11, K8.1, ORF19, ORF25, ORF26, ORF33, ORF37, and ORF63 increased with increasing age (Figure 3 B). Among seropositive individuals, antibody levels to the different KSHV and EBV proteins didn’t differ (Figure 3C) while antibody levels to the KSHV ORF73, K14 and ORF52 increased with increasing age (Figure 3D). As we have shown previously [27], IgG antibody levels to K8.1 were higher in individuals with detectable KSHV DNA in PBMCs (Figure 4A) and in oral fluids (Figure 4B). Additionally, in comparison to previous findings, IgG antibody levels to ORF65 and K10.5 were also higher in individuals with detectable KSHV DNA in PBMCs (Figure 4A) and oral fluids (Figure 4B). Furthermore, IgG antibody levels to ORF25 and ORF38 were higher in individuals with detectable KSHV DNA in oral fluids (Figure 4B).

Age was significantly associated with EBV detection in both oral fluids (P=0.025) and PBMCs (P=0.0089). On the other hand, both *P. falciparum* infection (detected by malaria rapid diagnostic tests-RDT) and sex were not associated with the detection of EBV in oral fluids or PBMCs (Table 2).

**Figure 5:**
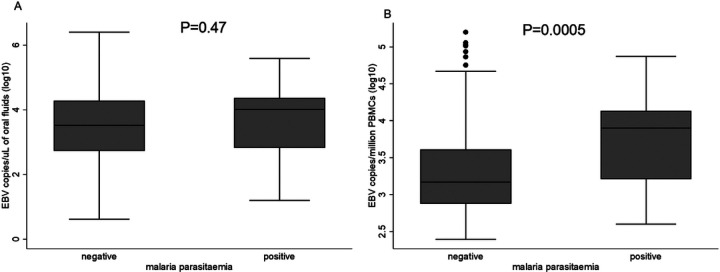
EBV viral copies in oral fluids (A) and PBMCs (B) by malaria parasitaemia status. P values obtained from a student T-test. Malaria parasitaemia was determined using Rapid diagnostic tests (RDT). EBV quantified in oral fluids and PBMCs using qPCR.

Among those with EBV in oral fluids, malaria and sex were not associated with EBV levels. However, among those with EBV in PBMCs, malaria was positively associated with EBV DNA levels. Individuals with malaria had higher levels of EBV DNA in PBMCs compared to individuals without malaria (adjusted regression coefficient 0.43, (0.15–0.71), P=0.002). Sex was not associated with levels of EBV DNA in PBMCs. Age group was associated with levels of EBV DNA in both oral fluids and PBMCs (Table 3 & Figure 5).

## Discussion

This study presents the following observations: (1) both in oral fluids and PBMCs, EBV is detected more frequently and in higher quantities compared to KSHV, as shown previously [25]; (2) both viruses are more likely to be detected in children’s oral fluids and PBMCs than among adults; (3) Individuals with KSHV in PBMCs are more likely to have EBV in PBMCs as well; (4) Infection with asymptomatic *P. falciparum* malaria is associated with higher EBV viral load in PBMCs; (5) in addition to IgG antibody levels to the KSHV K8.1, IgG antibody levels to KSHV ORF65 and K10.5 are higher in individuals with detectable KSHV in PBMCs and oral fluids while IgG antibodies to the KSHV ORF38 and ORF25 are higher in individuals with detectable KSHV in oral fluids only.

EBV is ubiquitous in all human populations with over 90% of adults infected [32] whereas KSHV is limited to specific geographic areas or high-risk populations, most notably in sub-Saharan Africa [33]. The findings suggest that EBV is more easily transmitted than KSHV. The mechanism leading to the difference in transmissibility between the two viruses is not known. This study and previous studies show that EBV DNA is more frequently detected in oral fluids and at higher levels than KSHV. This observation contributes to our understanding of the differences in transmission patterns between the two viruses. The difference cannot be solely explained by cell tropism because both KSHV and EBV infect several types of cells, some of which overlap. EBV infects B lymphocytes, epithelial cells, T lymphocytes, NK cells, monocytes, smooth muscle cells and follicular dendritic cells using CD21, HLA-II, integrins and EphA2 for attachment, internalization and entry [34]. KSHV infects endothelial cells, fibroblasts, monocytes, epithelial cells, B lymphocytes, macrophages and dendritic cells using HSPGs, DC-SIGN, EphAs and integrins for attachment and entry [35].

EBV and KSHV are more likely to be detected in children compared to adults. This might be attributed to a more recent infection with the virus. Viral control may have not been well established in children and could be developed over time as individuals age. Furthermore, the high burden of malaria infection in children could be driving viral reactivation of KSHV and EBV. We observed that individuals with detectable KSHV in PBMCs are more likely to have detectable EBV as well, we speculate that systemic factors affecting viral immune control including immunosuppression, Th2 skew, immune regulation, and immune cell dysfunction could affect the control of both viruses

Epidemiology studies have linked EBV and *P. falciparum* to Burkitt’s lymphoma [36]. Both EBV and *P. falciparum* upregulate AID expression while AID expression has been shown to contribute to c-MYC translocation and mutation [37–39]. c-MYC translocation is a hallmark of Burkitt lymphoma development [38]. Additionally, chronic exposure to *P. falciparum* has been shown to reactivate EBV, increasing the number of latently infected B cells with EBV [40]. Possibly *P. falciparum* impairs T cell immunity to EBV through immune suppression leading to EBV viral reactivation hence increasing the number of B cells infected by EBV. We have previously shown a similar association between *P. falciparum* infection and increased KSHV viral load [24]. The current finding that detection of EBV increases the risk of detecting KSHV in PBMCs, and the similar association of EBV and KSHV viral load in PBMCs with malaria infection, suggests that malaria could be affecting EBV and KSHV by causing immune dysfunction leading to viral reactivation of both viruses.

The strength of this study was the large sample size (over 800 individuals analysed) and the inclusion of males and females across the life course (3 to 89 years). Furthermore, all individuals analysed were HIV uninfected, so the impact of HIV on viral reactivation is not a concern. Although HIV has been shown to dramatically reactivate both viruses, in endemic regions, transmission of both viruses occurs in childhood before HIV acquisition for most individuals. The major weakness of this study is the cross-sectional design of the study.

## Conclusion

EBV is more frequently detected and at higher levels, both in PBMCs and oral fluids than in KSHV. Viral detection of both KSHV and EBV is more frequent in children compared to adults. This might partly be explained by the burden of *P. falciparum* infection in children and the recent viral infection. The mechanism through which *P. falciparum* affects both KSHV and EBV warrants further investigation.

## Figures and Tables

**Table 1:** Population characteristics and infection status

Age, mean (range)	36 (3–89) years
age groups (years)	3% (26/833)
3–5	11% (93/833)
6–12	10.5% (88/833)
13–17	10.7% (89/833)
18–25	16.6% (139/833)
26–35	14.5% (121/833)
36–45	14.5% (121/833)
46–55	8.8% (73/833)
56–65	10.6% (88/833)
66+	
Sex, males	49% (409/833)
Malaria parasitaemia^a^	4% (34/833)
EBV DNA levels in oral fluids (copies/uL)-median (IQR)	3,364 (557, 18,860)
KSHV DNA levels in oral fluids (copies/uL)-median (IQR)	401 (28, 3,921)
EBV DNA levels in PBMCs (copies/10^6^ cells)-median (IQR)	1566 (782, 4,378)
KSHV DNA levels in PBMCs (copies/10^6^ cells)-median (IQR)	203 (4, 620)
Individuals with detectable EBV in oral fluids	74% (607/824)
Individuals with detectable KSHV in oral fluids	24% (209/874)
Individuals with detectable EBV in PBMCs	46% (377/823)
Individuals with detectable KSHV in PBMCs	11% (94/869)

a asymptomatic malaria by rapid diagnostic test (RDT). Viral load detected using qPCR.

**Table 2:** Factors associated with EBV detection in oral fluids or PBMCs

Factor	Oral fluids			PBMCs		
	% with virus	OR (95% CI)*	P value	% with virus	OR (95% CI)*	P value
Sex						
Females	70% (297/422)	1	0.056	44% (183/420)	1	0.226
Males	77% (310/402)	1.36 (0.99, 1.86)		48% (194/403)	1.19 (0.90, 1.57)	
Age groups (years)						
3–17	82% (166/203)	1	0.025	50% (101/204)	1	0.0089
18–50	71% (282/397)	0.57 (0.37, 0.87)		40% (159/395)	0.70 (0.50, 0.99)	
51–89	71% (159/224)	0.57 (0.36, 0.91)		52% (117/224)	1.14 (0.78, 1.68)	
Malaria parasitaemia ^a^						
Negative	73% (580/790)	1	0.772	46% (361/789)	1	0.935
Positive	79% (27/34)	1.14 (0.48, 2.70)		47% (16/34)	1.03 (0.51, 2.08)	

* adjusted for age group, sex and malaria parasitaemia.

a asymptomatic malaria by rapid diagnostic test (RDT). Logistic regression modelling was done using STATA version 13. Viral load detected using qPCR.

**Table 3:** Factors associated with EBV viral load among individuals with detectable EBV in oral fluids or PBMCs

Factor	Oral fluids		PBMCs	
	coefficient (95% CI)*	P value	coefficient (95% CI)*	P value
Sex				
Females	ref	0.550	ref	0.348
Males	0.06 (−0.13, 0.24)		−0.05 (−0.16, 0.06)	
Age groups (years)				
3–17	ref	0.046	ref	0.0004
18–50	−0.23 (−0.45, −0.004)		−0.26 (−0.40, −0.12)	
51–89	−0.31 (−0.56, −0.05)		−0.08 (−0.22, 0.07)	
Malaria parasitaemia ^a^				
Negative	ref	0.826	ref	0.002
Positive	0.05 (−0.40, 0.50)		0.43 (0.15, 0.71)	

* adjusted for age group, sex and malaria parasitaemia.

a asymptomatic malaria by rapid diagnostic test (RDT). Logistic regression modelling was done using STATA version 13. Viral load detected using qPCR.

## Data Availability

All data analysed during this study are included in this published article as supplementary file 1 raw data
